# STRUMP-I: Structure-Based Machine Learning Approach to pMHC-I Binding Prediction Using Force Field Energy Features

**DOI:** 10.34133/csbj.0201

**Published:** 2026-08-11

**Authors:** Adam Voshall, Jeongjun Chae, Honglan Li, Junsu Ko, Naimur Rahman, Woongyang Park, Eunjung Alice Lee, Yoonjoo Choi

**Affiliations:** ^1^ Division of Genetics and Genomics, Boston Children’s Hospital, Boston, MA, USA.; ^2^ Department of Pediatrics, Harvard Medical School, Boston, MA, USA.; ^3^ Samsung Genome Institute, Samsung Medical Center, Seoul, Republic of Korea.; ^4^ Arontier Inc., Seoul, Republic of Korea.; ^5^ Department of Microbiology and Immunology, Chonnam National University Medical School, Hwasun, Republic of Korea.; ^6^ The National Immunotherapy Innovation Center, Hwasun, Republic of Korea.; ^7^ Geninus Inc., Seoul, Republic of Korea.; ^8^ Department of Health Science and Technology, Samsung Advanced Institute of Health Science and Technology, Sungkyunkwan University, Seoul, Republic of Korea.

## Abstract

The adaptive immune system monitors cellular integrity by recognizing short peptides from intracellular proteins presented on major histocompatibility complex class I (MHC-I) molecules, collectively termed peptide–MHC complexes (pMHC), enabling detection of foreign or mutated proteins. With the rising importance of immunotherapies targeting cancer neoantigens, accurately predicting which peptides bind to MHC alleles is critical. Current computational methods for pMHC-I binding prediction fall into sequence-based methods, which rely heavily on large training datasets, and structure-based methods that leverage structural modeling and pMHC binding energetics. Although sequence-based methods are widely used, their performance depends on the size and quality of the training data. Structure-based approaches, by contrast, can generalize better across diverse MHC alleles, but they traditionally depend on identifying a single global minimum-energy conformation, an assumption that may be inadequate for the promiscuous binding of MHC-I molecules. To address these limitations, we developed STRUMP-I (STRUcture-based pMHC Prediction for class I), a novel pMHC-I binding prediction tool that directly leverages a broad set of force-field-derived energy terms as machine learning features. In the standard benchmark set, STRUMP-I achieved performance comparable to state-of-the-art sequence-based models overall and showed a clear advantage for alleles with limited or imbalanced representation. Furthermore, STRUMP-I complemented sequence-based methods by removing method-specific false positives and improving precision, with a more favorable precision–recall tradeoff than AF-FT. These evaluations reinforced the value of STRUMP-I as a structure-informed prioritization method, particularly for underrepresented alleles and as a high-precision post-prediction filter.

## Introduction

The major histocompatibility complex (MHC) forms a complex with short peptides derived from endogenously expressed proteins, together known as the pMHC-I, which allows the adaptive immune system to monitor the internal functioning of a cell and survey for foreign or altered self-proteins [1]. The process involves proteasomal degradation, peptide transport, and loading into the MHC-I, which are then inspected by CD8^+^ T cells, leading to the proliferation of T cells that can bind to the peptide and the death of the cell presenting it. With the rising importance of immunotherapies targeting neoantigens in cancers [2] and the growing awareness of the importance of T cell immunity in vaccine design [3], accurately predicting which peptides will trigger an immune response is critical. Predicting the MHC-I binding of the peptide forms the core part of many neoantigen prediction pipelines [4]. However, as human leukocyte antigen (HLA) class I genes are among the most polymorphic genes in the human genome [5] and the number of known alleles continues to grow [6], this task remains extremely challenging across the vast diversity of possible MHC-I alleles. To address this, substantial computational efforts have been made to develop and refine prediction models that can effectively navigate the immense diversity of pMHC-I complexes.

Computational methods for predicting pMHC interactions fall into 2 broad categories: sequence-based and structure-based methods. In general, sequence-based methods have been widely used in practice since they are faster and more accurate than structure-based ones [7]. Nearly all the sequence-based methods recently developed use machine learning techniques to score the strength of the binding directly from the peptide and MHC sequences [8]. These methods often incorporate (pseudo-)sequence embeddings that represent key regions of the MHC molecules interacting with peptides. By leveraging large datasets of binding affinity and eluted ligand (EL) for the most common MHC alleles, the methods infer residue constraints at specific positions in the peptides and identify biologically meaningful motifs that interact with the binding pockets within the MHC binding groove [7]. However, the vast majority of MHC-I alleles have few or no known peptides to train on, limiting their generalizability for vaccine and cancer immunotherapy development [9]. To overcome this limitation, the current state-of-the-art methods, including NetMHCpan [10,11], MHCflurry [12], and HLAthena [13], specifically trained pan-allele models capable of predicting binding scores for MHC alleles absent from the training set, but the performance of these models on low abundance and unseen alleles still lags behind those alleles with better representation [14].

On the other hand, structure-based methods have been underdeveloped compared to sequence-based ones. These methods have primarily focused on accurately reconstructing the orientation and position of the bound peptide within the pMHC binding groove, referred to as the peptide binding mode [15]. While these methods typically optimize for binding energy, the scoring functions used in protein design methods such as Rosetta [16] act as correlates for binding affinity. Such approaches also allow the incorporation of modified or noncanonical residues within the peptide [17]. While these methods have underperformed the current state of the art sequence-based methods [18], they are less dependent on quality and quantity of the training data and generalize better to MHC alleles and peptides that are poorly represented or missing from the training set [19]. Recent methods have reduced the predictive performance gap either by using a per-position rather than global scoring scheme [20] or by leveraging the advancements made in ab initio protein folding using deep learning by training AlphaFold [21,22] to learn both structure and binding affinity of the pMHC [19,23].

Recent related work has extended structure-informed immune-recognition modeling beyond pMHC-I binding. STAG-LLM [24] integrates protein language models with computationally generated 3-dimensional (3D) structures for T cell receptor (TCR)–peptide HLA (pHLA) binding prediction, TCR-ESM [25] uses protein language embeddings to predict TCR–pMHC binding, and recent benchmarking of TCR–pMHC modeling web servers [26] highlights both the value and remaining limitations of structural modeling for T cell recognition. Complementary workflows in immunopeptidomics and vaccine design, including machine-learning filters for mass-spectrometry-based immunopeptide identification, Immunolyser 2.0, and recent computational vaccine development reviews, further illustrate the broader computational pipeline surrounding antigen discovery [27–29].

Here, we present STRUMP-I (STRUcture-based pMHC Prediction for class I), a pMHC-I binding prediction tool that combines template-based structural modeling, molecular relaxation, FoldX-based energy decomposition, and LightGBM classification to discriminate binders from nonbinders. Existing structure-based pMHC-I scoring approaches generally use a modeled or docked pMHC-I conformation to compute a global energy, docking, or structural score that acts as a proxy for binding. This strategy compresses a complex interaction into a single minimum-energy or near-minimum-energy summary. However, the promiscuous nature of pMHC-I binding indicates that multiple local interaction patterns can be compatible with stable binding, so a single global score may not capture the full information contained in the modeled complex. STRUMP-I was designed around a different representation: Rather than treating the final structure as a source of one binding-energy score, it extracts multiple force-field-derived energetic descriptors and template-quality features and uses them directly as supervised machine-learning features.

## Materials and Methods

### Datasets

#### IEDB dataset

All human MHC ligand assay results were exported from IEDB (http://iedb.org) [30] and filtered following the strategy outlined by [13]. Only pMHC-I pairs with quantitative binding-affinity measurements were retained. Specifically, we retained entries measured using “purified MHC/direct/radioactivity/dissociation constant KD”, “purified MHC/direct/fluorescence/half maximal effective concentration (EC50)”, or “cellular MHC/direct/fluorescence/half maximal effective concentration (EC50)” assay types. We removed peptides that were present multiple times in the database when the difference between the maximum and minimum value for the log-transformed affinity [1 − log(nM)/log(50,000)] was >0.2. For the remaining pMHC-I pairs, peptides with an affinity ≤500 nM were considered binders, and those with an affinity >500 nM were considered nonbinders. After filtering, there was a total of 115,644 pairs from this dataset.

#### HLAthena datasets

We combined the IEDB dataset with the MHC-I ELs from mono-allelic cell lines published alongside HLAthena [13]. Because these peptides were derived from cell lines that expressed only a single MHC allele, all peptides are considered binders for the allele expressed in the cell line they were detected in. We filtered any noncanonical alleles and peptides, leaving a total of 173,142 pMHC-I pairs. After merging the datasets, we removed any redundant pMHC-I pairs. The final IEDB + HLAthena dataset contained 288,639 pMHC-I pairs, consisting of 207,529 binders and 81,110 nonbinders.

#### Public neoantigen datasets

We downloaded the TESLA [31] dataset via the SYNAPSE consortium. As with the IEDB and HLAthena datasets, we considered any peptide with a binding affinity ≤500 nM as a binder. The TESLA source file contained 500 pMHC-I pairs; 491 analysis-ready pairs remained after removing rows with missing binding affinity, collapsing duplicate allele–peptide pairs, and requiring successful predictions from all benchmarked methods. The PRIME [32] dataset was taken from the original publication. Peptides annotated as “Random” were considered nonbinders, and peptides from all other studies of origin were considered binders. Rows with missing labels and control peptides were removed before evaluation. The final PRIME benchmark contained 7,690 pMHC-I pairs. For the immunogenicity predictions, the ground truth was taken from the datasets. The PRIME % rank for the corresponding MHC-I allele was calculated using PRIME2.0 [33].

#### VACINUS neoantigen dataset from cancer patients

The VACINUS neoantigen candidates were collected from 33 cancer patients (6 with colorectal cancer, 2 with melanoma, 14 with hepatocellular carcinoma, and 11 with gastric cancer) recruited at Asan Medical Center (IRB number 2022-0263) [34]. All procedures were carried out in accordance with relevant guidelines and regulations. We selected highly expressed nonsynonymous or indel mutations using whole transcriptome sequencing (WTS) data. The selection criteria included the variant allele frequency (VAF) > 0, read counts > 10 at the transcript level, and the sum of transcripts per million (TPM) calculated based on genes > log(0.4). We predicted cleavage products using NetChop and MHCflurry. Cleaved peptide products were initially screened using NetMHCpan and MHCflurry. Peptides from the intersection of these prediction sets were experimentally validated using the NeoScreen MHC/Peptide Binding Assays (Immunitrack ApS, Copenhagen, Denmark). The fluorescent peptide dextramers were synthesized by Immudex, Copenhagen, Denmark. The final dataset consists of 120 pMHC-I pairs, of which 101 are binders and 19 are nonbinders.

#### Dataset overlap analysis

To assess potential data leakage, we quantified overlap among datasets at 3 levels: exact pMHC-I pair overlap, peptide-sequence overlap irrespective of allele, and allele-level overlap. Exact pMHC-I overlap was defined as an identical normalized allele–peptide pair. Same-peptide overlap was defined as the same peptide sequence appearing in both datasets regardless of allele, and peptide-only overlap was defined as same-peptide overlap after excluding exact pMHC-I matches. For the IEDB + HLAthena development benchmark, overlap was computed among the train, validation, and test partitions generated from the same analysis-ready rows used for model evaluation. For external benchmarking, overlap was computed between the full IEDB + HLAthena dataset used to train the final STRUMP-I model and each external evaluation set. Results are reported in Table S1.

### STRUMP-I pipeline

#### Generation of model structure and energy calculation

A total of 826 template pMHC Protein Data Bank (PDB) structures were downloaded from the IMGT database, including both pMHC-I and TCR–pMHC-I complexes. Following download, we extracted the 181 amino acids comprising the G-domain of the MHC-I molecule, which contains the peptide-binding groove. Given a query peptide and MHC-I sequence, STRUMP-I first restricts the template search to pMHC-I complex structures with the same peptide length as the query peptide. Among length-matched templates, the most similar MHC-I allele is identified by aligning the query MHC-I sequence to each template MHC-I sequence using the BLOSUM62 scoring matrix. The HLA BLOSUM ratio was defined as the BLOSUM62 alignment score between the query and template MHC-I sequences divided by the BLOSUM62 self-alignment score of the query MHC-I sequence. If multiple candidate templates have the same MHC-I similarity score, STRUMP-I then selects the template with the most similar peptide using the PAM30 scoring matrix. The peptide PAM ratio was defined analogously as the PAM30 alignment score between the query and template peptide sequences divided by the PAM30 self-alignment score of the query peptide.

Following template selection, STRUMP-I uses the BuildModel module from FoldX to replace the template peptide and any nonmatching MHC-I residues with the query peptide and MHC-I sequence. This step generates a query-specific pMHC-I model while preserving the experimentally resolved peptide-binding mode of the selected template. The BLOSUM62/PAM30 ranking procedure is therefore an integral part of STRUMP-I template selection because template choice determines the mutated pMHC-I model, the relaxed structure, and the FoldX-derived feature vector. The mutated PDB structure is then relaxed with the Tinker molecular dynamics package, version 8.9.5 [35], using the AMBER99sb parameter set with the GB-HPMF implicit solvent model. This relaxation step reduces steric clashes and unfavorable conformations introduced during in silico mutation.

To generate the final model and energy features, the relaxed structure is further optimized using FoldX until the total energy no longer improves. FoldX side-chain optimization and complex-energy analysis are then performed on the final optimized pMHC-I structure. The final energetics of the structure are calculated using the AnalyzeComplex module of FoldX, treating the peptide and MHC-I molecule as interacting partners. The resulting STRUMP-I feature vector includes the total pMHC-I interaction energy and component FoldX energy terms, including van der Waals interactions, electrostatics, polar and hydrophobic solvation terms, hydrogen-bond terms, entropy terms, and clash-related terms. These FoldX-derived energy descriptors are combined with template-quality descriptors, including the BLOSUM62 and PAM30 alignment scores and ratios, as input features for classification. The complete feature list is provided in Table S2.

#### Model training and classification

For model development, the IEDB + HLAthena dataset was merged with STRUMP-I structural-quality and FoldX energy-feature files using the allele and peptide sequence as keys. We removed timing columns and nonfeature identifiers, filtered out structural models with final FoldX energy greater than or equal to zero, and removed duplicate allele–peptide pairs after merging. The remaining analysis-ready rows were used for model training, validation, and testing.

For benchmarking STRUMP-I on the IEDB + HLAthena dataset, we first split the analysis-ready dataset into 80% train/validation and 20% test sets using stratification on the binary binding label. We then split the train/validation set into 80% training and 20% validation sets, again stratified by the binary binding label, yielding effective train/validation/test proportions of 64%/16%/20%. The random seed was fixed at 42 for both splitting steps. Feature scaling was performed by fitting the STRUMP-I scaler on the training set and applying the fitted scaler to the validation and test sets.

We built STRUMP-I using the Python implementation of LightGBM, version 4.0.0 [36]. We tuned the hyperparameters for the model using version 2.3.0 of the Optuna optimization library for Python for 10,000 rounds with pruning [37]. The hyperparameters used to train the final model are presented in Table S3. Unless otherwise noted, STRUMP-I prediction scores greater than or equal to 0.5 were classified as binders. Following benchmarking on the IEDB + HLAthena development split, we retrained STRUMP-I using the full analysis-ready IEDB + HLAthena dataset and the same hyperparameters to generate the final model for benchmarking on the TESLA, PRIME, and VACINUS neoantigen datasets. We tested feature importance for this final model using version 0.43.0 of the SHAP (SHapley Additive exPlanations) Python library [38,39].

#### Model performance comparison

The performance of STRUMP-I was compared against 3 sequence-based pMHC-I binding prediction tools and 2 structure-based binding prediction tools. The 3 sequence-based tools were NetMHCpan version 4.1 [10], HLAthena [13], and MHCflurry version 2.0 [12]. Predictions from all methods were merged by allele and peptide sequence so that performance metrics were calculated on the same peptide–MHC-I rows for all benchmarked tools. Allele names were normalized to a common format before merging where required by the corresponding tool output files.

NetMHCpan was evaluated using 2 outputs: the eluted-ligand rank score, %Rank_EL, and the predicted binding affinity in nM. We used the recommended NetMHCpan binding cutoff of %Rank_EL ≤ 0.5 and also evaluated the affinity-based cutoff of predicted affinity ≤ 500 nM. Because lower NetMHCpan rank and affinity values indicate stronger predicted binding, these scores were directionally reversed for AUROC (area under the receiver operating characteristic curve) and PRAUC (area under the precision–recall curve) calculations so that larger continuous values consistently corresponded to stronger predicted binding.

MHCflurry was evaluated using both its predicted affinity and affinity percentile outputs. We used the affinity percentile cutoff recommended for classification, Affinity percentile ≤ 2%, and also evaluated the affinity-based cutoff of predicted affinity ≤ 500 nM. As with NetMHCpan, lower MHCflurry affinity and percentile values indicate stronger predicted binding, so these continuous scores were directionally reversed before calculating AUROC and PRAUC.

HLAthena was evaluated using its mass spectrometry-based intrinsic score (MSi) presentation score and percentile-rank MSi score. HLAthena does not report a predicted binding affinity in nM; therefore, in place of an affinity cutoff, we used MSi ≥ 0.5 as a score-based classification threshold. We also evaluated the recommended percentile-rank threshold, prank.MSi ≤ 0.2. For continuous-score metrics, MSi was used directly because larger values indicate stronger predicted presentation, whereas prank.MSi was directionally reversed because lower percentile-rank values indicate stronger predicted presentation.

AlphaFold-FineTune (AF-FT) [19] was benchmarked as the published AF-FT workflow rather than as a newly retrained or re-engineered derivative model. All pMHC PDB files were downloaded from the PDB website in December 2023, and candidate templates were identified for each input pMHC pair using the AF-FT sequence-similarity procedure based on BLOSUM62. For each pMHC-I pair, the logit-transformed predicted-aligned-error summary score produced by AF-FT was used as the continuous prediction score. AUROC and PRAUC for AF-FT were computed from the continuous AF-FT score and therefore do not depend on the binary classification threshold.

We also evaluated SwiftMHC [40] as an additional structure-based comparator on the subset of examples compatible with the publicly available SwiftMHC inference model. Because the released SwiftMHC model currently supports only HLA-A*02:01 9-mer peptides, this comparison was restricted to pMHC-I pairs with normalized allele HLA-A*02:01 and peptide length 9. SwiftMHC was therefore not included in the full-dataset benchmark and was instead reported as a scope-limited comparison. SwiftMHC predictions were evaluated using the same binary labels and performance metrics used for the other methods, where applicable.

#### Performance metrics

All methods were evaluated as binary classifiers and as continuous-scoring predictors. For binary classification, true positives (TP), false positives (FP), true negatives (TN), and false negatives (FN) were computed after applying each method-specific threshold. Accuracy was defined as

$$\text{Accuracy}=\frac{\text{TP}+\text{TN}}{\text{TP}+\text{FP}+\text{TN}+\text{FN}}.$$ (1)

Sensitivity, also referred to as recall, was defined as

$$\text{Sensitivity}=\text{Recall}=\frac{\text{TP}}{\text{TP}+\text{FN}}.$$ (2)

Specificity was defined as

$$\text{Specificity}=\frac{\text{TN}}{\text{TN}+\text{FP}}.$$ (3)

Precision, also referred to as positive predictive value (PPV), was defined as

$$\text{Precision}=\mathrm{PPV}=\frac{\mathrm{TP}}{\text{TP}+\text{FP}}.$$ (4)

The F1 score was defined as

$$F1=2\times \frac{\text{Precision}\times \text{Recall}}{\text{Precision}+\text{Recall}}.$$ (5)

Matthews correlation coefficient (MCC) was defined as

$$\mathrm{MCC}=\frac{\left(TP\times TN\right)-\left(FP\times FN\right)}{\sqrt{\begin{array}{c} \left(\text{TP}+\text{FP}\right)\times \left(\text{TP}+\text{FN}\right)\\ \times \left(\text{TN}+\text{FP}\right)\times \left(\text{TN}+\text{FN}\right) \end{array}}}$$ (6)

For continuous-score evaluation, we calculated AUROC and PRAUC. All performance metrics were computed using Scikit-learn.

For allele-level analyses, performance metrics were calculated separately for each allele. Allele-level performance was then compared with the number of training examples for that allele and with the allele-specific positive ratio, defined as the number of binder examples divided by the total number of binder and nonbinder examples for that allele in the training set.

For combined-predictor analyses, a pMHC-I pair was considered positive for a tool combination only if all tools in the combination classified the pair as a binder. These intersection-based calls were used to evaluate whether adding STRUMP-I or AF-FT to sequence-based predictors improved precision by removing method-specific false-positive predictions.

#### Runtime benchmarking

To quantify computational cost, we benchmarked STRUMP-I and the full-dataset comparator methods on a common set of 100 randomly sampled pMHC-I pairs containing 9-mer peptides. Runtime was measured as wall-clock time per pMHC-I pair on a single workstation with an AMD Ryzen 7 7700X CPU, 64 GiB RAM, and an NVIDIA GeForce RTX 3060 GPU. Sequence-based predictors were run on the central processing unit (CPU). AF-FT was run on the graphics processing unit (GPU). STRUMP-I was benchmarked in both CPU and GPU configurations, where GPU acceleration was applied to the Tinker minimization step. For STRUMP-I, we additionally recorded the runtime of template selection, in silico mutation, Tinker minimization, FoldX optimization and energy analysis, and feature extraction separately. Runtime and failure-rate results are reported in Table S5.

## Results

### Development of the STRUMP-I model

**Fig. 1.**
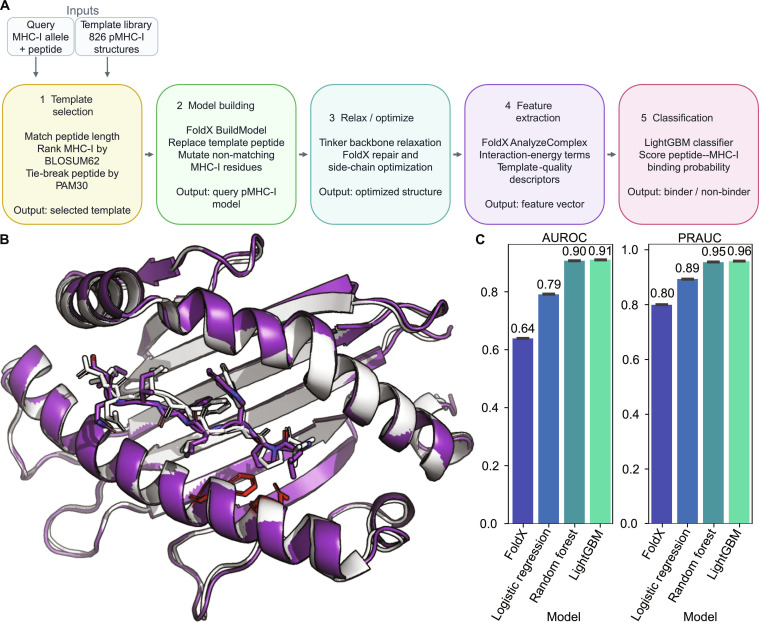
Overview of the STRUMP-I binding prediction pipeline. (A) Simplified STRUMP-I workflow showing the 5 major stages of the method: template selection from the query MHC-I allele and peptide, FoldX-based model building, structural relaxation and optimization, FoldX energy-feature extraction, and LightGBM-based binding classification. (B) Superimposition of the structures for a query sequence with HLA-B*51:01 (white) and matched template sequence with HLA-B*52:01 (purple) after in silico mutation and backbone relaxation. Red side chains represent mutations in the MHC. (C) AUROC and PRAUC scores for initial tests of different machine-learning classifiers on the test set from the IEDB + HLAthena training dataset, showing the average of 100 replicates of 90/10 train/test splits.

In order to leverage the structural information about pMHC binding, we developed a machine-learning classifier, STRUMP-I, that utilizes force-field energy values as features calculated from protein homology-model structure. An overview of the STRUMP-I workflow is presented in Fig. 1A. Briefly, for a given peptide length and MHC type, STRUMP-I identifies the most similar MHC template structure from the known structure database, and a model structure is built by in silico mutating differing positions. The mutated structure is then backbone-relaxed and optimized for the AMBER99sb force field [41] with Head-Gordon’s hydrophobic potential of mean force with the generalized Born model (GB-HPMF) [42] using the Tinker molecular dynamics package [35] to remove potential steric clashes due to the mutations (Fig. 1B). After the relaxation, side chains were optimized with the FoldX force field [43] and their energy terms were directly utilized as features. Apart from the force-field energy terms, other quantifiable measures such as the sequence similarities between structure template and input sequence for both MHC and peptide were also included in the feature set.

To develop the classifier, we built train, validation, and test sets consisting of a combination of the IEDB database and EL data used in the development of HLAthena [13]. After filtering, this dataset contained 288,639 pMHC-I pairs, consisting of 207,529 binders and 81,110 nonbinders (Data file S1). We first compared the scaled FoldX interaction energy between the peptide and MHC-I with machine-learning classifiers trained on the full set of STRUMP-I features listed in Table S2. The scaled FoldX interaction energy alone was a poor predictor of binding (Fig. 1C and Fig. S1A). Among the machine-learning classifiers tested, logistic regression, random forest, and LightGBM were evaluated using 100 random seeds with a 90/10 train/test split. LightGBM achieved the highest performance by both AUROC and PRAUC and was selected for further development (Fig. 1C). The performance of the LightGBM model was further improved with hyperparameter tuning using Optuna [37]. The final model shows clear discrimination between binders (binding affinity < 500 nM) and nonbinders (binding affinity ≥ 500 nM) (Fig. S1B). Based on a SHAP [38,39] analysis, many of the most important features were nonbonded energy terms, including the overall interaction energy, solvation hydrophobicity and polarity, and electrostatic interactions (Fig. S2). Template-quality features were also important, including the alignment scores and the HLA BLOSUM ratio, defined as the BLOSUM62 alignment score between the query and template MHC-I sequences divided by the BLOSUM62 self-alignment score of the query MHC-I sequence. This indicates that identifying an appropriate structural template is critical for predicting pMHC-I binding.

### STRUMP-I shows stable prediction even on alleles with low representation

We evaluated STRUMP-I against 3 widely used sequence-based pMHC binding prediction tools: NetMHCpan v4.1, MHCflurry v2.0, and HLAthena. For the sequence-based tools, we evaluated 2 classification criteria where available, as described in Materials and Methods. We also compared STRUMP-I with the AlphaFold-based peptide binding prediction method AF-FT. We first evaluated all methods using the test set constructed from the IEDB [30] plus HLAthena datasets [13] (IEDB + HLAthena), which consists of 38,302 binders and 9,061 nonbinders from 115 alleles.

**Fig. 2.**
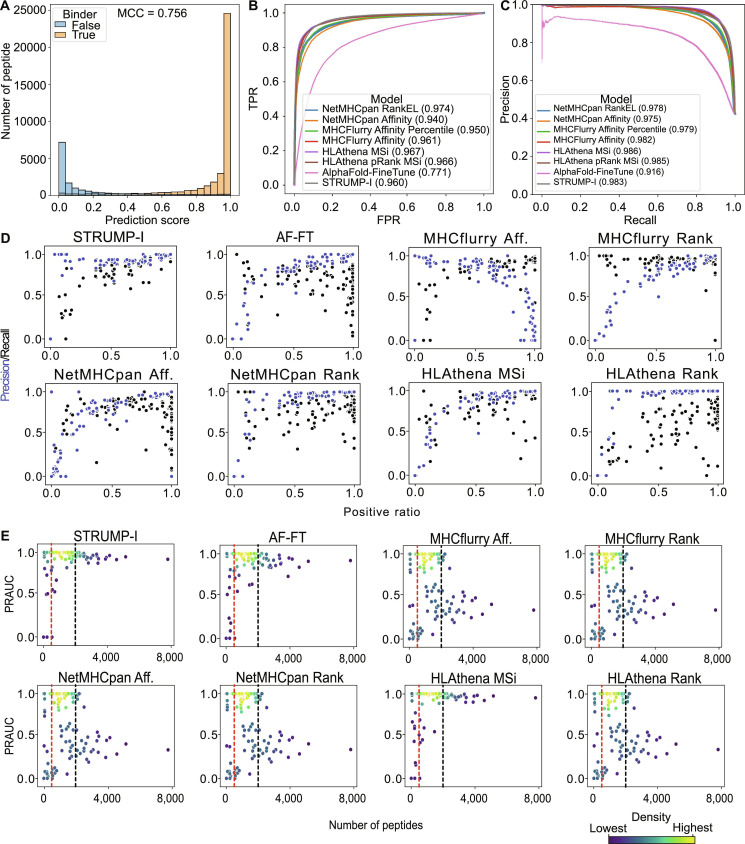
Performance of STRUMP-I on the IEDB + HLAthena test set. (A) STRUMP-I prediction scores for binders (orange) and nonbinders (blue). (B) ROC curves for each method tested on the IEDB + HLAthena test set. TPR, true positive rate; FPR, false positive rate. (C) Precision–recall curves for each method tested on the IEDB + HLAthena test set. (D) Relationship between the training-set positive ratio and allele-level precision (blue) or recall (black). The positive ratio is defined as the number of positive peptide–MHC-I pairs divided by the total number of positive and negative pairs for that allele in the training data. (E) Scatterplots showing allele-level PRAUC as a function of the number of peptide–MHC-I pairs for each allele in the IEDB + HLAthena training set. Points are colored by density, with blue representing low density and yellow representing high density. Alleles with fewer than 2,000 training examples, denoted by the black dashed line, constitute the average-representation set. Alleles with fewer than 500 training examples, denoted by the red dashed line, constitute the low-representation set.

We also assessed potential overlap among the IEDB + HLAthena development partitions. No exact pMHC-I pairs were shared between the training, validation, and test sets, and no exact-overlap label conflicts were observed (Table S1). Same-peptide and allele-level overlap were present, as expected for a stratified random split rather than a peptide- or allele-held-out split. Therefore, the IEDB + HLAthena benchmark evaluates generalization to unseen pMHC-I pairs, but not necessarily to entirely unseen peptides or unseen alleles. Within this dataset, STRUMP-I recovered the second highest number of binders (35,149) behind only MHCflurry (36,275 or 35,480, depending on binding measure used) while retaining up to half as many nonbinders (2,056 versus 3,928 for MHCflurry affinity percentile), giving STRUMP-I an overall performance very comparable to the sequence-based methods with a PRAUC of 0.983 and MCC of 0.756, compared to a PRAUC of 0.982 and MCC of 0.781 for MHCflurry, a PRAUC of 0.986 and MCC of 0.778 for HLAthena, and a PRAUC of 0.974 and MCC of 0.603 for NetMHCpan (Fig. 2A to C) with similar scores across other summary metrics (Table S6). AF-FT underperformed on this dataset compared to STRUMP-I and all of the sequence-based methods with a PRAUC of 0.916 and MCC of 0.422. This performance is nearly identical on the validation set used for hyperparameter tuning (Table S7).

These differences in performance are most pronounced for alleles that are less abundant or that have less balanced representation within the training dataset. For alleles appearing in the external benchmark datasets, Table S8 lists the corresponding training-set size and positive ratio, defined as the number of positive examples divided by the total number of pairs for each allele; the overall distributions across training alleles are shown in Fig. S3. Methods heavily dependent on the underlying data distribution typically show an arch-shaped distribution in performance metrics, characterized by peaks in precision and recall at intermediate positive ratios (balanced representation of positive and negative examples). Performance often drops off at extremes of the positive ratio, highlighting sensitivity to dataset balance. Less data-dependent methods demonstrate relatively stable or level performance across the full range of positive ratios, indicated by the absence of clear peaks or troughs, with precision and recall values distributed evenly from low to high positive ratios. The affinity predictions for NetMHCpan, MHCflurry, and MSi score for HLAthena all show this arch-shaped pattern, with a high precision but low recall for alleles with a high positive ratio and a low precision and high recall for alleles with a low positive ratio (Fig. 2D). The rank-based scores for each sequence-based method had better precision across positive ratios, but NetMHCpan RankEL and HLAthena MSi Rank in particular had inconsistent recall even for alleles with high positive ratios. Only STRUMP-I maintains high precision across the full range, at a moderate cost to recall (Fig. 2D).

When the most represented alleles were excluded, and we measured the performance on only alleles with <2,000 peptides present in the training set (average representation; Fig. S3), the performance of most tools increased slightly with STRUMP-I, increasing to a PRAUC and MCC of 0.995 and 0.828, comparable to HLAthena (PRAUC: 0.994, MCC: 0.822) and ahead of MHCflurry (0.987, 0.702), NetMHCpan (0.987, 0.581), and AF-FT (0.949, 0.359) (Fig. 2E, Fig. S4, and Table S9). Notably, when we measured the performance on only those alleles with <500 peptides in the training set (low representation; Fig. S3), the performance of all methods, except NetMHCpan using predicted affinity, dropped (Fig. S4 and Table S10). This drop in performance was most pronounced in MHCflurry (PRAUC: 0.952, MCC: 0.732), HLAthena (0.932, 0.5), and AF-FT (0.934, 0.279). In contrast, STRUMP-I’s performance remains consistently high (0.989, 0.881).

This pattern is consistent with sequence-based models being more sensitive to allele-specific data abundance and label balance. When alleles have few examples or highly imbalanced positive-to-negative ratios, sequence-based models may learn less reliable allele-specific motifs or thresholds. STRUMP-I instead uses template-derived structural features and FoldX energy terms that are not tied as directly to the number of examples for a particular allele, which may explain why its performance is more stable for low-representation alleles. While this does not imply that STRUMP-I is unaffected by template quality or structural-modeling errors, it suggests that its errors are less driven by allele-specific data scarcity.

### Benchmarking STRUMP-I with neoantigen datasets

**Fig. 3.**
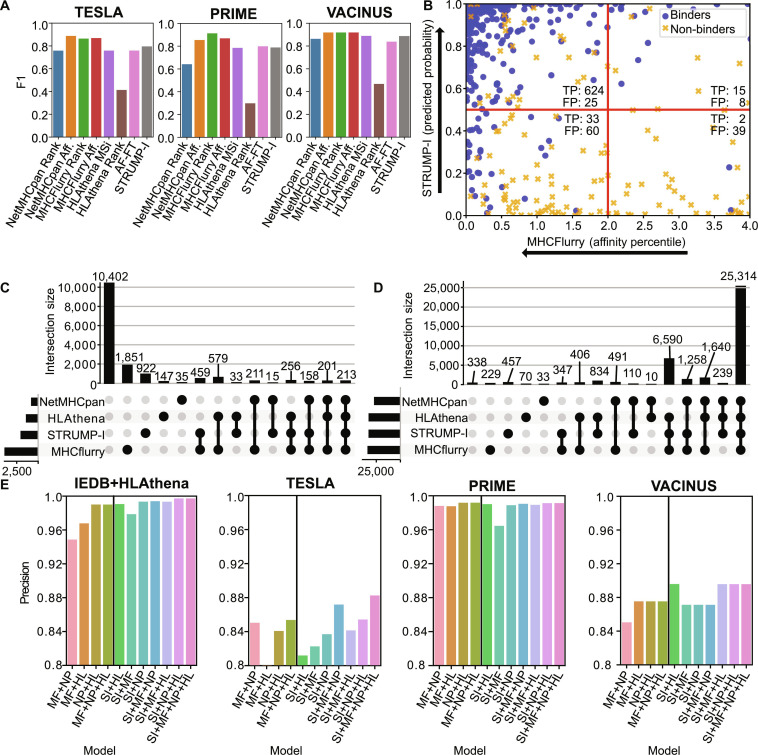
Binding prediction performance on external benchmark datasets and precision gains from combining predictors. (A) Bar plots showing F1 scores on the TESLA, PRIME, and VACINUS datasets. (B) Scatterplot comparing MHCflurry affinity-percentile scores and STRUMP-I predicted binding probabilities for a randomly sampled subset of IEDB + HLAthena test-set peptide–MHC-I pairs with predictions from both methods. Binders are shown as blue points and nonbinders as orange x’s. Red lines indicate the classification thresholds for each method. For MHCflurry, lower affinity-percentile scores indicate stronger predicted binding, whereas for STRUMP-I, higher predicted probabilities indicate stronger predicted binding. Peptides in the upper-left quadrant are predicted to bind by both tools. (C and D) UpSet plots showing nonbinders (C) or binders (D) predicted as binders by each tool using the classification thresholds described in Materials and Methods. (E) Precision scores for intersections of predictions from multiple tools across the benchmark datasets, with the *y* axis zoomed to precision values above 0.8 for readability. Bars to the left of the vertical line show sequence-based method combinations alone, and bars to the right show combinations that include STRUMP-I. Quantitative values are provided in the corresponding supplementary tables. MF, MHCflurry; NP, NetMHCpan; HL, HLAthena; SI, STRUMP-I.

Following benchmarking on the IEDB + HLAthena dataset, we retrained STRUMP-I on the full dataset for benchmarking 2 additional public neoantigen datasets, TESLA [31] and PRIME [32]. Because the external benchmarks were evaluated using the STRUMP-I model trained on the full IEDB + HLAthena dataset, we separately quantified overlap between the full training set and each external dataset. TESLA and VACINUS had no exact pMHC-I overlap and no peptide-level overlap with IEDB + HLAthena. PRIME contained 1,019 exact pMHC-I overlaps among 7,690 evaluated rows (13.3%), plus 67 additional same-peptide/different-allele rows; after removing exact overlaps, 6,671 PRIME rows remained (Table S1). We therefore interpret PRIME as a partially overlapping external benchmark. These datasets predominantly (PRIME) or entirely (TESLA) consist of average or overrepresented alleles (Table S8). When applied to these additional benchmark datasets, performance was more dataset-dependent. STRUMP-I showed intermediate performance on TESLA, but lower overall performance than the sequence-based methods on PRIME. STRUMP-I’s overall performance on the TESLA dataset, with a PRAUC of 0.866, fell between the HLAthena outputs and AF-FT on the low end (PRAUCs of 0.831 to 0.838) and NetMHCpan and MHCflurry on the high end (PRAUCs of 0.929 and 0.937) (Tables S11 and S12). The corresponding thresholded F1 scores are shown in Fig. 3A. In particular, the PRIME result should be interpreted cautiously: STRUMP-I achieved a PRAUC of 0.846, whereas the other evaluated methods ranged from 0.953 to 0.988. While STRUMP-I performs strongly for low-representation alleles in the IEDB + HLAthena-derived benchmark, its performance on TESLA is intermediate and its overall performance on PRIME is lower than that of the sequence-based tools, consistent with the difference in performance on overrepresented alleles (Tables S11 to S13).

To address the availability of an additional structure-based comparator, we also evaluated SwiftMHC on the subset of benchmark examples compatible with its released inference model, namely, HLA-A*02:01 9-mer peptides (Table S4). This subset analysis contained 1,886 IEDB + HLAthena test examples, 891 PRIME examples, 107 TESLA examples, and only 3 VACINUS examples. SwiftMHC performed well on the IEDB + HLAthena HLA-A*02:01 9-mer subset (PRAUC = 0.967, AUROC = 0.967, MCC = 0.814) and on the PRIME subset (PRAUC = 1.000, AUROC = 0.995, MCC = 0.920). However, performance was less robust on TESLA, where SwiftMHC showed high binder recall but poor nonbinder recall, resulting in a lower MCC of 0.315. The VACINUS subset contained only 3 compatible peptides, all binders, making threshold-independent metrics and nonbinder performance undefined. Thus, SwiftMHC provides a useful restricted-scope structure-based comparison, but it does not evaluate the pan-allele or underrepresented-allele setting targeted by STRUMP-I.

### Integrating STRUMP-I with sequence-based prediction methods improves precision

STRUMP-I achieves high predictive accuracy but intrinsically requires more computational time than other methods. To explicitly assess this computational cost, we benchmarked STRUMP-I and the full-dataset comparator methods on a set of 100 randomly sampled pMHC-I pairs (9-mer peptides only) using a single workstation (AMD Ryzen 7 7700X, 64 GiB RAM, NVIDIA GeForce RTX 3060). The 3 sequence-based methods required a few seconds per pair on the CPU (NetMHCpan, 0.06 s; MHCflurry, 4.08 s; HLAthena, 6.07 s), while the structure-based AF-FT required 104.6 s per pair on the GPU (Table S5).

STRUMP-I can be executed in CPU-only mode when GPU resources are unavailable or in GPU-accelerated mode for faster structure generation. On the CPU, STRUMP-I required an average of 22.1 min per pair—distributed across in silico mutation (2.5 min), Tinker minimization (14.3 min), and FoldX energy analysis (5.3 min)—with template selection and energy extraction requiring less than 0.1 s. When utilizing the GPU, the Tinker minimization step is reduced to 10 s, bringing the total per-pair runtime down to 7.9 min.

Because of this computational overhead, STRUMP-I is most practically deployed when integrated with sequence-based binding prediction methods within neoantigen prediction pipelines to improve overall precision. When comparing prediction scores between sequence-based methods and STRUMP-I, we found that STRUMP-I and the sequence-based predictors more often agreed for true binders, whereas false-positive predictions were more often method-specific (Fig. 3B). While many false-positive predictions are tool-specific and are filtered out by any combination of tools, the inclusion of STRUMP-I with the sequence-based methods further removes false-positive predictions for every combination of sequence-based tools (Fig. 3C and D). These results suggest that STRUMP-I and the sequence-based methods make partially orthogonal errors. Sequence-based tools can classify peptides as binders when they resemble learned allele-specific motifs, whereas STRUMP-I additionally requires the modeled peptide–MHC-I complex to have a favorable structural-energy profile. Requiring agreement between methods therefore removes many method-specific false-positive predictions and increases precision, although this intersection strategy reduces recall. Consequently, every combination containing STRUMP-I has a higher precision than the corresponding combination without STRUMP-I, reaching as high as 0.991 on the IEDB + HLAthena dataset and 0.990 on the PRIME dataset when combining the predictions of every tool (Fig. 3E).

To determine whether this precision improvement reflected a general effect of adding any structure-based predictor, or was more specific to STRUMP-I, we repeated the same intersection analysis using AF-FT as the structure-based filter (Table S14). AF-FT combinations also increased precision relative to the corresponding sequence-based predictions, but the gains were consistently smaller than those obtained with STRUMP-I and were accompanied by larger recall losses. Across all sequence-based combinations on the IEDB + HLAthena test set, STRUMP-I increased precision by an average of 0.024 compared with 0.010 for AF-FT, while the average recall loss was 0.062 for STRUMP-I and 0.200 for AF-FT. For example, combining MHCflurry with STRUMP-I increased precision from 0.903 to 0.975 while maintaining recall of 0.866, whereas combining MHCflurry with AF-FT increased precision to 0.944 but reduced recall to 0.672. Similarly, for the 3-way sequence-based consensus of MHCflurry, NetMHCpan, and HLAthena, STRUMP-I increased precision from 0.985 to 0.993 with recall of 0.653, whereas AF-FT increased precision only to 0.986 with recall of 0.561. These results indicate that structure-based filtering can improve precision, but STRUMP-I provides a more favorable precision–recall tradeoff than AF-FT in this setting.

### Prediction of unseen neoantigen candidate peptides

In addition to public datasets, we used the VACINUS neoantigen dataset, a set of 120 noncontrol pMHC-I pairs selected from 129 neoantigen candidates from 33 cancer patients (6 colorectal cancer, 2 melanoma, 14 hepatocellular carcinoma, and 11 gastric cancer) and spanning 6 overrepresented and 1 average-representation alleles (Table S15) [34]. NetChop and MHCflurry were used to predict protein cleavage sites, and NetMHCpan and MHCflurry were used to pre-filter peptides for binding potential before peptide synthesis and binding confirmation with Immunitrack ApS (Copenhagen, Denmark). Based on how this dataset was generated, both NetMHCpan and MHCflurry have a recall of 1, but each also produced 38 false-positive binder predictions, leading to a precision of 0.85 and PRAUC values ranging from 0.928 to 0.960 (Table S16). The corresponding F1 scores are shown in Fig. 3A, and precision values for tool combinations are shown in Fig. 3E. Consistent with the other benchmark datasets, STRUMP-I further reduces the number of false-positive predictions in this dataset down to 28 at a moderate cost to recall, increasing the precision to 0.870 (Fig. 3E and Table S16) with a PRAUC of 0.928. This performance makes STRUMP-I the second most precise method on this dataset, comparable to HLAthena (precision of 0.876 and PRAUC of 0.907) and ahead of AF-FT (precision of 0.864 and PRAUC of 0.905).

## Discussion

Here, we presented STRUMP-I, a novel structure-based pMHC class I binding prediction pipeline, along with the VACINUS dataset consisting of 101 binders and 19 nonbinders [34]. Our results show that structure-based methods, including STRUMP-I and AF-FT, can provide useful and complementary information to sequence-based predictors, although their relative performance varies across datasets. In our comprehensive benchmark set, STRUMP-I was less sensitive to allele representation and label imbalance than other methods. This relative robustness may arise because STRUMP-I does not rely only on allele-specific sequence motifs inferred from examples for a given allele.

On external datasets, STRUMP-I showed intermediate performance on TESLA and lower overall performance than sequence-based tools on PRIME. Several factors may contribute to the lower PRIME performance. PRIME predominantly contains average- or overrepresented alleles, a setting in which sequence-based pan-allele tools are expected to perform well due to the abundance of the training data. It is worth noting that PRIME is a neoepitope-oriented benchmark whose labels and negative examples differ from direct biochemical binding-affinity measurements, which may favor methods that capture sequence motifs or presentation-related signals beyond structural pMHC-I energetics. Our overlap analysis also showed that PRIME is partially overlapping with the full IEDB + HLAthena training set, so it should not be interpreted as a fully independent external benchmark.

During our analyses, we compared STRUMP-I to another recent structure-based pMHC binding prediction method, AF-FT. STRUMP-I and AF-FT performed comparably on TESLA and PRIME; however, STRUMP-I substantially outperformed AF-FT on the test data derived from the IEDB and HLAthena datasets. AF-FT, SwiftMHC, and STRUMP-I represent different structure-based prediction strategies with different native workflows. For this reason, we benchmarked AF-FT and SwiftMHC as existing comparator methods rather than re-engineering their template-selection or classification steps. STRUMP-I’s BLOSUM62/PAM30 template selection and LightGBM classifier are therefore features of the STRUMP-I method itself, while AF-FT performance reflects the published AF-FT workflow based on its logit-PAE-derived score. While the computational requirements for STRUMP-I to generate the structure of the pMHC are greater than the sequence-based methods, the improved performance on poorly represented alleles in the training data and generation of pMHC structures for downstream analyses can justify the additional computational time, especially when STRUMP-I is used as a post-prediction filter for sequence-based tools as part of a neoantigen prediction pipeline.

The SwiftMHC subset analysis further highlights the diversity of current structure-based pMHC-I prediction approaches. SwiftMHC is computationally efficient and performed well for some HLA-A*02:01 9-mer subsets, but its current allele- and length-specific scope prevents evaluation of the low-representation and pan-allele settings that motivate STRUMP-I. Together with AF-FT, these results suggest that structural information can be useful for pMHC-I prediction, but that the practical value of a structure-based method depends on both predictive performance and coverage across alleles and peptide lengths.

The reduction of false positives observed when STRUMP-I is combined with sequence-based predictors likely reflects the orthogonality of structural and sequence-based errors. In this use case, efficient sequence-based methods can rapidly screen a patient’s full proteome for potential binders, and STRUMP-I can then apply a structural-energy filter to remove candidates that resemble binders by sequence but do not form favorable modeled pMHC-I interactions. This strategy creates a higher-confidence set of candidate peptides before experimental validation, but it also involves an explicit precision–recall tradeoff: Requiring agreement between multiple tools increases precision while reducing sensitivity.

The AF-FT combination analysis reinforces this interpretation but shows that not all structure-based filters provide the same tradeoff. AF-FT also increased precision when intersected with sequence-based predictions, but the precision gains were smaller and recall losses were larger than for STRUMP-I (Table S14).

The principal strengths and limitations of STRUMP-I are as follows.

**Strengths:**

1. STRUMP-I is less dependent on allele-specific training data than sequence-based predictors and maintains strong performance for alleles with limited or imbalanced representation.

2. When combined with sequence-based tools, STRUMP-I improves prediction precision by removing method-specific false-positive predictions, making it well suited as a secondary filter in large-scale screening pipelines.

3. STRUMP-I also produces a 3D pMHC-I model, which can support downstream analyses of peptide conformation, solvent exposure, immunogenicity, and TCR recognition.

**Limitations:**

1. Although STRUMP-I is less dependent on training data, it remains dependent on the availability and quality of suitable structural templates. Continued advances in pMHC structure prediction may reduce this limitation.

2. STRUMP-I is substantially more computationally demanding than sequence-based methods. This cost may be mitigated by replacing the most time-consuming modeling steps with more efficient GPU-accelerated implementations.

3. For well-represented alleles, STRUMP-I shows no consistent standalone advantage over established sequence-based predictors. Its most practical use in this setting is therefore as a complementary structural filter rather than as a replacement for rapid sequence-based screening.

4. STRUMP-I remains affected by dataset bias and label noise because it is trained on available public binding-affinity and eluted-ligand datasets.

5. The current model predicts pMHC-I binding and does not directly model antigen processing, peptide abundance, TCR recognition, or immunogenicity.

Our results indicate that STRUMP-I is particularly useful for alleles with limited representation in training data and for increasing precision when used as a post-prediction filter. The lower relative performance on PRIME and intermediate performance on TESLA suggest that external benchmark performance depends on dataset composition, allele representation, and label-generation strategy. We therefore interpret STRUMP-I as a complementary structure-based prioritization tool rather than a universal replacement for sequence-based pMHC-I binding predictors. Moving forward, the structural outputs generated by STRUMP-I present promising opportunities for future research, particularly in integrating structural features such as solvent accessibility, peptide flexibility, and detailed atomic interactions into predictive models for immunogenicity and TCR specificity.

## Data Availability

Source code and trained models are available at https://github.com/yoonjoolab/STRUMP-I.

## References

[1] Blum JS, Wearsch PA, Cresswell P. Pathways of antigen processing. Annu Rev Immunol. 2013;31:443–473. 23298205 10.1146/annurev-immunol-032712-095910 PMC4026165

[2] Schumacher TN, Schreiber RD. Neoantigens in cancer immunotherapy. Science. 2015;348(6230):69–74. 25838375 10.1126/science.aaa4971

[3] Heitmann JS, Bilich T, Tandler C, Nelde A, Maringer Y, Marconato M, Reusch J, Jäger S, Denk M, Richter M, et al. A COVID-19 peptide vaccine for the in duction of SARS-CoV-2 T cell immunity. Nature. 2022;601(7894):617–622. 34814158 10.1038/s41586-021-04232-5 PMC8791831

[4] Xie N, Shen G, Gao W, Huang Z, Huang C, Fu L. Neoantigens: Promising tar gets for cancer therapy. Signal Transduct Target Ther. 2023;8(1):9. 36604431 10.1038/s41392-022-01270-x PMC9816309

[5] Mungall AJ, Palmer SA, Sims SK, Edwards CA, Ashurst JL, Wilming L, Jones MC, Horton R, Hunt SE, Scott CE, et al. The DNA sequence and analysis of human chromo some 6. Nature. 2003;425(6960):805–811. 14574404 10.1038/nature02055

[6] Barker DJ, Maccari G, Georgiou X, Cooper MA, Flicek P, Robinson J, Marsh SGE. The IPD-IMGT/HLA database. Nucleic Acids Res. 2023;51(D1):D1053–D1060. 36350643 10.1093/nar/gkac1011 PMC9825470

[7] Nielsen M, Andreatta M, Peters B, Buus S. Immunoinformatics: Predicting peptide–MHC binding. Annual Rev Biomed Data Sci. 2020;3(1):191–215. 37427310 10.1146/annurev-biodatasci-021920-100259 PMC10328453

[8] Mei S, Li F, Leier A, Marquez-Lago TT, Giam K, Croft NP, Akutsu T, Smith AI, Li J, Rossjohn J, et al. A comprehensive review and performance evaluation of bioinformatics tools for HLA class I peptide-binding prediction. Brief Bioinform. 2020;21(4):1119–1135. 31204427 10.1093/bib/bbz051 PMC7373177

[9] Bravi B, Tubiana J, Cocco S, Monasson R, Mora T, Walczak AM. RBM-MHC: A semi-supervised machine learning method for sample-specific prediction of antigen presentation by HLA-I alleles. Cell Syst. 2021;12(2):195–202.e9. 33338400 10.1016/j.cels.2020.11.005 PMC7895905

[10] Reynisson B, Alvarez B, Paul S, Peters B, Nielsen M. NetMHCpan-4.1 and NetMHCIIpan-4.0: Improved predictions of MHC antigen presentation by concur rent motif deconvolution and integration of MS MHC eluted ligand data. Nucleic Acids Res. 2020;48(W1):W449–W454. 32406916 10.1093/nar/gkaa379 PMC7319546

[11] Nilsson JB, Kaabinejadian S, Yari H, Kester MGD, van Balen P, Hildebrand WH, Nielsen M. Accurate prediction of HLA class II anti gen presentation across all loci using tailored data acquisition and refined machine learning. Sci Adv. 2023;9(47): Article eadj6367. 38000035 10.1126/sciadv.adj6367 PMC10672173

[12] O’Donnell TJ, Rubinsteyn A, Laserson U. MHCflurry 2.0: Improved pan-allele prediction of MHC class I presented peptides by incorporating anti gen processing. Cell Syst. 2020;11(1):418–419. 33091335 10.1016/j.cels.2020.09.001

[13] Sarkizova S, Klaeger S, le PM, Li LW, Oliveira G, Keshishian H, Hartigan CR, Zhang W, Braun DA, Ligon KL, et al. A large peptidome dataset improves HLA class I epitope prediction across most of the human population. Nat Biotechnol. 2020;38(2):199–209. 31844290 10.1038/s41587-019-0322-9 PMC7008090

[14] Glynn E, Ghersi D, Singh M. Toward equitable major histocompatibility complex binding predictions. Proc Natl Acad Sci USA. 2025;122(8): Article e2405106122. 39964728 10.1073/pnas.2405106122 PMC11874272

[15] Perez MAS, Cuendet MA, Rohrig UF, Michielin O, Zoete V. Structural prediction of peptide-MHC binding modes. Methods Mol Biol. 2022;2405:245–282. 35298818 10.1007/978-1-0716-1855-4_13

[16] Rohl CA, Strauss CE, Misura KM, Baker D. Protein structure prediction using Rosetta. Methods Enzymol. 2004;386, 383:66–93. 10.1016/S0076-6879(04)83004-0 15063647

[17] Bloodworth N, Barbaro NR, Moretti R, Harrison DG, Meiler J. Rosetta FlexPepDock to predict peptide-MHC binding: An approach for non-canonical amino acids. PLOS ONE. 2022;17(12): Article e0275759. 36512534 10.1371/journal.pone.0275759 PMC9746977

[18] Antunes DA, Abella JR, Devaurs D, Rigo MM, Kavraki LE. Structure based methods for binding mode and binding affinity prediction for peptide MHC complexes. Curr Top Med Chem. 2018;18(26):2239–2255. 30582480 10.2174/1568026619666181224101744 PMC6361695

[19] Motmaen A, Dauparas J, Baek M, Abedi MH, Baker D, Bradley P. Peptide binding specificity prediction using fine-tuned protein structure prediction networks. Proc Natl Acad Sci USA. 2023;120(9): Article e2216697120. 36802421 10.1073/pnas.2216697120 PMC9992841

[20] Conev A, Devaurs D, Rigo MM, Antunes DA, Kavraki LE. 3pHLA score improves structure-based peptide HLA binding affinity prediction. Sci Rep. 2022;12(1):10749. 35750701 10.1038/s41598-022-14526-x PMC9232595

[21] Jumper J, Evans R, Pritzel A, Green T, Figurnov M, Ronneberger O, Tunyasuvunakool K, Bates R, Žídek A, Potapenko A, et al. Highly accurate protein structure prediction with AlphaFold. Nature. 2021;596(7873):583–589. 34265844 10.1038/s41586-021-03819-2 PMC8371605

[22] Abramson J, Adler J, Dunger J, Evans R, Green T, Pritzel A, Ronneberger O, Willmore L, Ballard AJ, Bambrick J, et al. Accurate structure prediction of biomolecular interactions with AlphaFold 3. Nature. 2024;630(8016):493–500. 10.1038/s41586-024-07487-w PMC11168924 38718835

[23] Mikhaylov V, Levine AJ. Accurate modeling of peptide-MHC structures with AlphaFold. bioRxiv. 2023. 10.1101/2023.03.06.531396 PMC10872456 38113889

[24] Slone JK, Zhang M, Jiang P, Montoya A, Bontekoe E, Rausseo BN, Reuben A, Kavraki LE. STAG-LLM: Predicting TCR-pHLA binding with protein language models and computationally generated 3D structures. Comput Struct Biotechnol J. 2025;27:3885–3896. 41542080 10.1016/j.csbj.2025.09.004 PMC12800378

[25] Yadav S, Vora DS, Sundar D, Dhanjal JK. TCR-ESM: Employing protein language embeddings to predict TCR-peptide-MHC binding. Comput Struct Biotechnol J. 2024;23:165–173. 38146434 10.1016/j.csbj.2023.11.037 PMC10749252

[26] Le HN, Vaz de Freitas M, Antunes DA. Strengths and limitations of web servers for the modeling of TCRpMHC complexes. Computational and structural. Comput Struct Biotechnol J. 2024;23:2938–2948. 39104710 10.1016/j.csbj.2024.06.028 PMC11298609

[27] Wei F, Kouro T, Nakamura Y, Ueda H, Iiizumi S, Hasegawa K, Asahina Y, Kishida T, Morinaga S, Himuro H, et al. Enhancing mass spectrometry-based tumor immunopeptide identification: Machine learning filter leveraging HLA bind ing affinity, aliphatic index and retention time deviation. Comput Struct Biotechnol J. 2024;23:859–869. 38356658 10.1016/j.csbj.2024.01.023 PMC10864759

[28] Munday PR, Krishna SSG, Fehring J, Croft NP, Purcell AW, Li C, Braun A. Immunolyser 2.0: An advanced computational pipeline for comprehensive analysis of immunopeptidomic data. Comput Struct Biotechnol J. 2025;29:296–304. 41209766 10.1016/j.csbj.2025.10.007 PMC12590289

[29] Hashim O, Dimier-Poisson I. Computational vaccine development against protozoa. Comput Struct Biotechnol J. 2025;27:2386–2393. 40529181 10.1016/j.csbj.2025.06.011 PMC12172979

[30] Vita R, Mahajan S, Overton JA, Dhanda SK, Martini S, Cantrell JR, Wheeler DK, Sette A, Peters B. The immune epitope database (IEDB): 2018 update. Nucleic Acids Res. 2019;47(D1):D339–D343. 30357391 10.1093/nar/gky1006 PMC6324067

[31] Wells DK, van Buuren MM, Dang KK, Hubbard-Lucey VM, Sheehan KC, Campbell KM, Lamb A, Ward JP, Sidney J, Blazquez AB, Rech AJ. Key parameters of tumor epitope immunogenicity revealed through a consortium approach improve neoantigen prediction. Cell. 2020;183(3):818–834.e13. 33038342 10.1016/j.cell.2020.09.015 PMC7652061

[32] Schmidt J, Smith AR, et al. Prediction of neo-epitope immunogenicity reveals TCR recognition determinants and provides insight into immunoediting. Cell Rep Med. 2021;2(2): Article 100194. 33665637 10.1016/j.xcrm.2021.100194 PMC7897774

[33] Gfeller D, Schmidt J, Croce G, Guillaume P, Bobisse S, Genolet R, Queiroz L, Cesbron J, Racle J, Harari A. Improved predictions of antigen presentation and TCR recognition with MixMHCpred2.2 and PRIME2.0 reveal potent SARS-CoV-2 CD8^+^ T-cell epitopes. Cell Syst. 2023;14:72–1483.e5. 36603583 10.1016/j.cels.2022.12.002 PMC9811684

[34] Kim SH, Lee BR, Kim SM, Kim S, Kim MS, Kim J, Lee I, Kim HS, Nam GH, Kim IS, et al. The identification of effective tumor-suppressing neoantigens using a tumor-reactive TIL TCR-pMHC ternary complex. Exp Mol Med. 2024;56:1461–1471. 38866910 10.1038/s12276-024-01259-2 PMC11263684

[35] Rackers JA, Wang Z, Lu C, Laury ML, Lagardère L, Schnieders MJ, Piquemal JP, Ren P, Ponder JW. Tinker 8: Software tools for molecular design. J Chem Theory Comput. 2018;14:5273–5289. 30176213 10.1021/acs.jctc.8b00529 PMC6335969

[36] Ke G, Meng Q, Finley T, Wang T, Chen W, Ma W, Ye Q, Liu TY. Light GBM: A highly efficient gradient boosting decision tree. In: *Advances in neural information processing systems. Vol. 30*. Curran Associates Inc.; 2017. p. 3146–3154.

[37] Akiba T, Sano S, Yanase T, Ohta T. and Koyama M. Optuna: A next generation hyperparameter optimization framework. arXiv. 2019. 10.48550/arXiv.1907.10902

[38] Lundberg SM, Lee SI. A unified approach to interpreting model predictions. *Adv Neural Inf Process Syst*. 2017;**30**.

[39] Lundberg SM, Erion G, Chen H, DeGrave A, Prutkin JM, Nair B, Katz R, Himmelfarb J, Bansal N, Lee SI. From local explanations to global understanding with explainable AI for trees. Nat Mach Intell. 2020;2(1):56–67. 32607472 10.1038/s42256-019-0138-9 PMC7326367

[40] Baakman CAB, Crocioni G, Geng C, Rademaker DT, Frühbuß D, Aarts YJM, Xue LC. A high-speed attention network for MHC-bound peptide identification and 3D modeling. Cell Rep Methods. 2026;6(4): Article 101364. 41923631 10.1016/j.crmeth.2026.101364 PMC13106973

[41] Ponder JW, Case DA. Force fields for protein simulations. Adv Protein Chem. 2003;66:27–85. 14631816 10.1016/s0065-3233(03)66002-x

[42] Lin MS, Fawzi NL, Head-Gordon T. Hydrophobic potential of mean force as a solvation function for protein structure prediction. Structure. 2007;15(6):727–740. 17562319 10.1016/j.str.2007.05.004

[43] Schymkowitz J, Borg J, Stricher F, Nys R, Rousseau F, Serrano L. The FoldX web server: An online force field. Nucleic Acids Res. 2005;33(2 Suppl):W382–W388. 15980494 10.1093/nar/gki387 PMC1160148

