# Supplementary material for: STRUMP-I: Structure-Based Machine Learning Approach to pMHC-I Binding Prediction Using Force Field Energy Features
Source: Comput Struct Biotechnol J. 2026 Aug 11;35(1):0201. doi: 10.34133/csbj.0201 (PMC13457900; doi:10.34133/csbj.0201)

Supplementary Information

STRUMP-I: Structure-based machine learning approach to pMHC-I binding prediction using force field energy features

Adam Voshall[1,2,†], Jeongjun Chae[3,†], Honglan Li[4], Junsu Ko[4], Woongyang Park[3,5,6], Eunjung Alice Lee[1,2], and Yoonjoo Choi[7,8].

[†]These authors contributed equally to this work

[1]Division of Genetics and Genomics, Boston Children's Hospital, Boston, MA, USA, [2]Department of Pediatrics, Harvard Medical School, Boston, MA, USA
[3]Samsung Genome Institute, Samsung Medical Center, Seoul, Republic of Korea
[4]Arontier Inc., Seoul, Republic of Korea
[5]Geninus Inc., Seoul, Republic of Korea
[6]Department of Health Science and Technology, Samsung Advanced Institute of Health Science and Technology, Sungkyunkwan University, Seoul Republic of Korea
[7]Department of Microbiology and Immunology, Chonnam National University Medical School, Hwasun, Republic of Korea
[8]The National Immunotherapy Innovation Center, Hwasun, Republic of Korea

*Woongyang Park, Eunjung Alice Lee, and Yoonjoo Choi.
Email: woongyang@skku.edu (W.P.), ealice.lee@childrens.harvard.edu (E.A.L), or kalicuta@gmail.com (Y.C.)

Supplementary Tables (Table S1-S12) are provided in a separate spreadsheet file.

Figure S1: Density plots of binding affinity in the test set. White dashed line represents a binding affinity 500 nM used as a threshold to define binders and non-binders. A) Binding affinity and interaction energy calculated by FoldX. B) Binding affinity and prediction score from STRUMP-I.

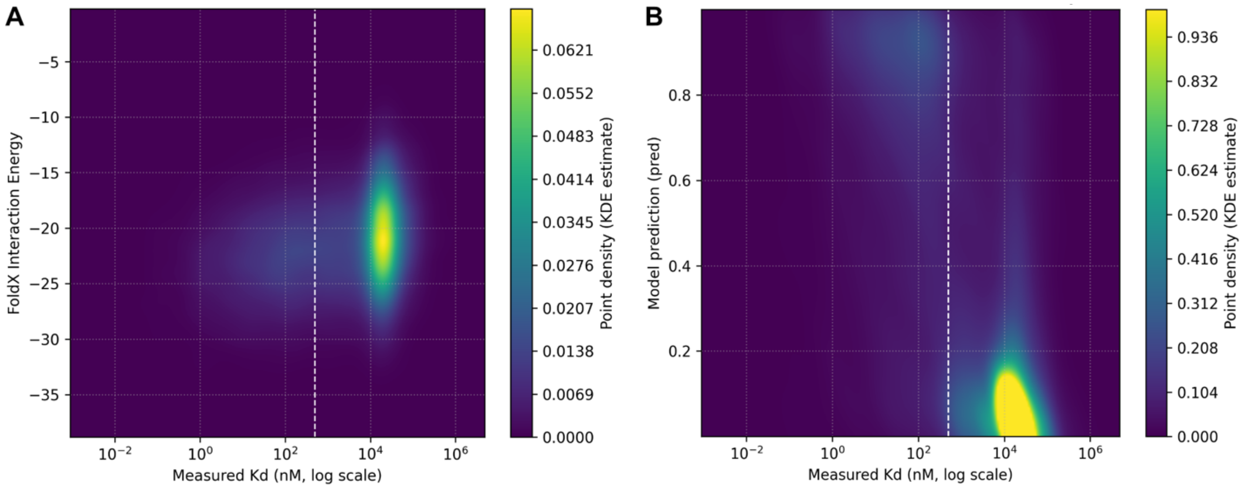

Figure S2: SHAP summary values for the top used for STRUMP-I binding prediction on the in-house cancer dataset. For a description of the features, see Table S1. The features are sorted by the magnitude of the SHAP value to highlight the overall most important features driving predictions. Left) Barplot showing the mean absolute value for the relative impact on the model output. Right) Beeswarm plot where red and blue dots indicate peptides with higher or lower than median values for the feature, respectively. The distance from the center line indicates the relative impact of the feature on the final prediction score for that peptide.

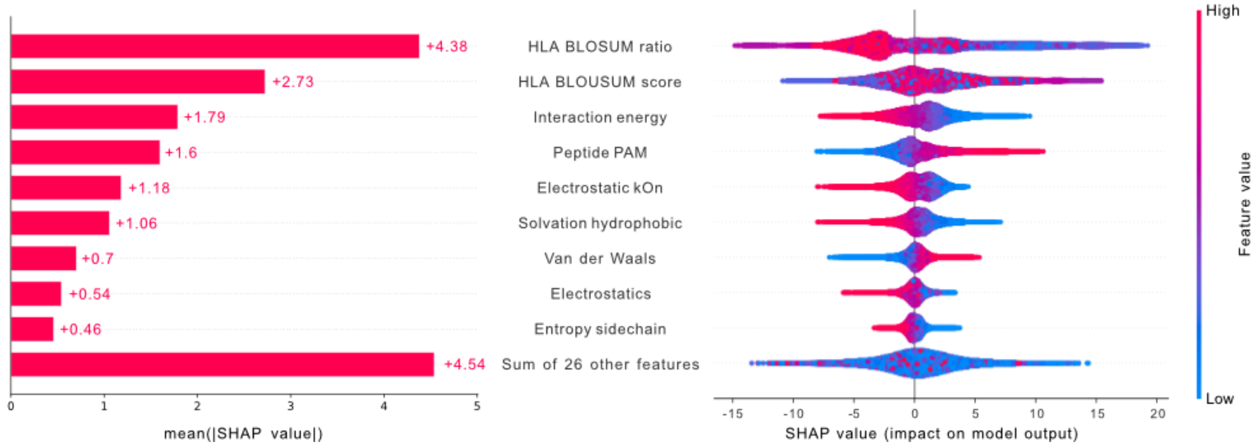

Figure S3: Histograms for number of examples per allele in the training set. A) Total number of peptides per allele. Red dashed line – 500 peptides per allele threshold. Alleles to the left of the line are classified as "Low represention". Black dashed line – 2000 peptides per allele. Alleles to the right of the line are classified as "Over represention". Alleles between the lines are classified as "Average representation". B) Positive ratio (percent of positive examples) per allele.

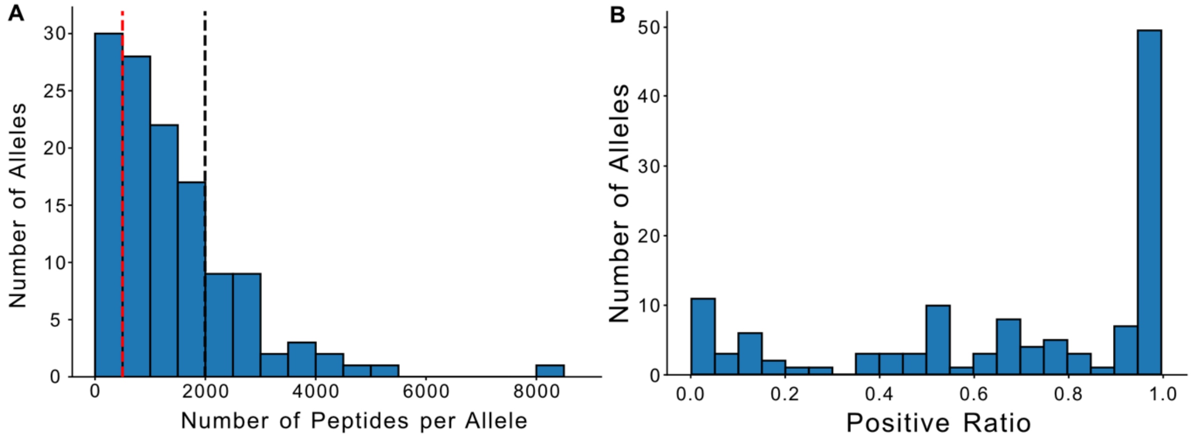

Figure S4: Method performance on Over, Average, and Low representation alleles. Performance measured by Matthew's Correlation Coefficient (MCC) (top) and PRAUC (bottom). Over representation are alleles with >= 2,000 pMHC pairs in the training set. Average representation are all alleles with < 2,000 pMHC pairs in the training set. Low representation are all alleles with < 500 pMHC pairs in the training set. The line represents equal performance on alleles in each category. Methods under the line show decreased performance on the over representation or low representation alleles.

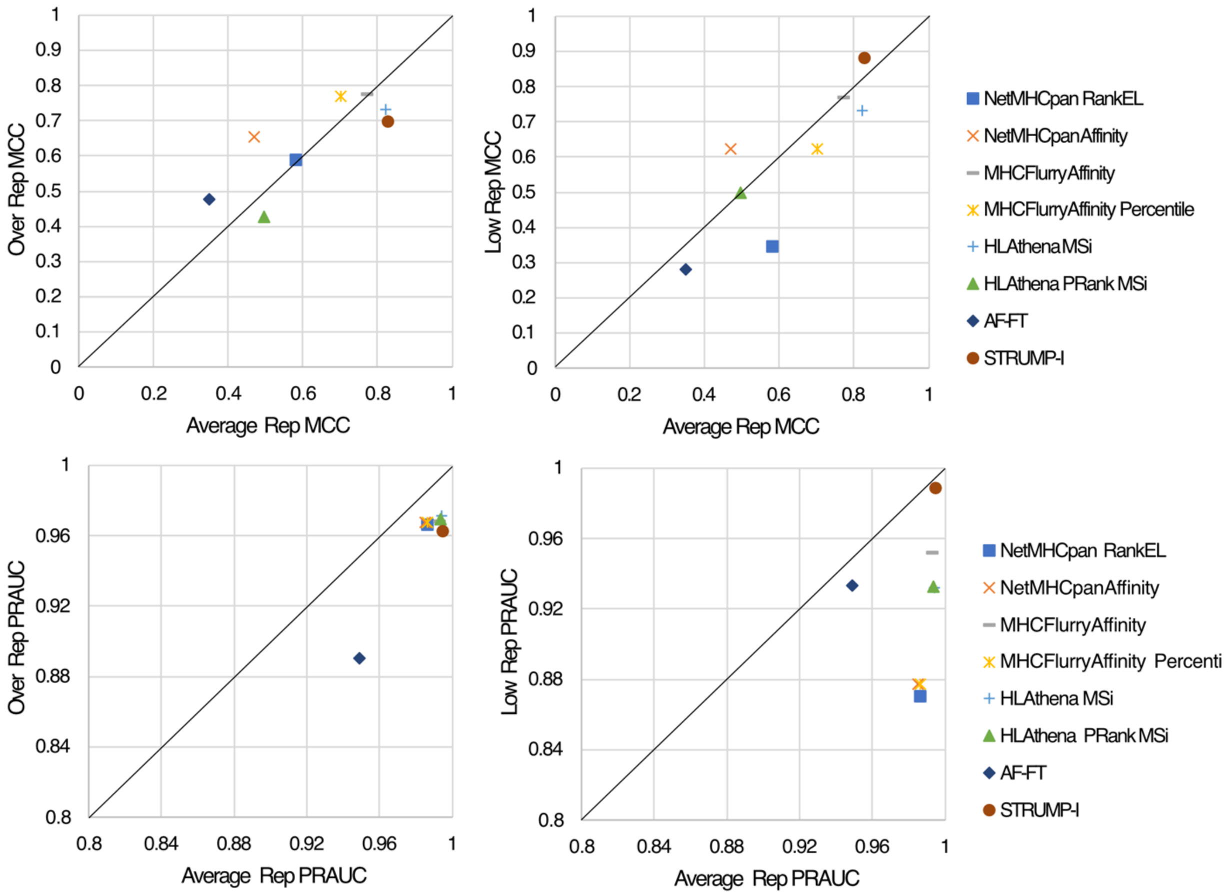

Supplement: Supplementary file 1 [file csbj.0201.f1.zip › SI_CSBJ-rev.pdf]
